# Role of MicroRNA Profile Modifications in Hepatitis C Virus-Related Mixed Cryoglobulinemia

**DOI:** 10.1371/journal.pone.0062965

**Published:** 2013-05-01

**Authors:** Elisa Fognani, Carlo Giannini, Alessia Piluso, Laura Gragnani, Monica Monti, Patrizio Caini, Jessica Ranieri, Teresa Urraro, Elisa Triboli, Giacomo Laffi, Anna Linda Zignego

**Affiliations:** Center for Systemic Manifestations of Hepatitis Viruses (MASVE), Department of Experimental and Clinical Medicine, University of Florence, Florence, Italy; University of Cincinnati College of Medicine, United States of America

## Abstract

Hepatitis C virus infection is closely related to lymphoproliferative disorders (LPDs), including mixed cryoglobulinemia (MC) and some lymphomas. Modification of the expression of specific microRNAs (miRNAs) has been associated with different autoimmune diseases and/or LPDs. No data exist about the modifications in miRNA expression in HCV-associated LPDs. The aim of this study was to analyze the expression levels of a panel of miRNAs previously associated with autoimmune/LPDs in a large population of HCV patients with and without MC or non-Hodgkin’s lymphoma (NHL), to identify potential markers of evolution of HCV infection. PBMC expression of miR-Let-7d, miR-16, miR-21, miR-26b, miR-146a and miR-155 was evaluated by real-time PCR in 167 HCV patients (75 with MC [MC-HCV], 11 with HCV-associated NHL [NHL-HCV], 81 without LPD [HCV]) and in 35 healthy subjects (HS). A significant increase in miR-21 (*p*<0.001), miR-16 (*p*<0.01) and miR-155 (*p*<0.01) expression was detected in PBMCs from only NHL patients whereas a significant decrease in miR-26b was detected in both MC and NHL subjects (*p*<0.01) when compared to HS and HCV groups. A restoration of miR-26b levels was observed in the post-treatment PBMCs of 35 HCV-MC patients experiencing complete virological and clinical response following antiviral therapy. This study, for the first time, shows that specific microRNAs in PBMC from HCV patients who developed MC and/or NHL are modulated differently. The specific, reversible downregulation of miR-26b strongly suggests the key role it plays in the pathogenesis of HCV-related LPDs and its usefulness as a biomarker of the evolution of HCV infection to these disorders.

## Introduction

Mixed cryoglobulinemia (MC) is both an autoimmune and lymphoproliferative disorder (LPD) characterized by circulating immune complexes named cryoglobulins (CGs) and composed of polyclonal IgGs (including anti-HCV Ig) and mono- or polyclonal IgM with rheumatoid factor (RF) activity, sustained by the clonal expansion of RF B cells [1], [2], [3], [4]. This is a benign, but pre-lymphomatous condition whose clinical manifestations -the so-called MC syndrome- are secondary to systemic vasculitis of the small/medium vessels [5], [6], [7]. MC is strongly associated with hepatitis C virus (HCV) infection: 80–90% of MC patients are HCV positive (in Italy >95%) and from 40 to 60% of HCV patients have circulating cryoglobulins, with 5–30% of these latter patients having a MC *syndrome*. MC is frequently characterized by bone marrow and/or liver infiltrates closely resembling non-Hodgkin’s Lymphoma (NHL) [5], therefore, it was hypothesized that HCV may be involved in the pathogenesis of B cell-NHL as well [8], [9]. This hypothesis was substantiated by several observations, including the significantly high prevalence of HCV infection in NHL patients in several studies [10], as well as the resolution or prevention of the lymphatic neoplasia after effective antiviral therapy [11], [12]. Therefore, since HCV infects about 200 million individuals worldwide, the number of patients at risk for LPD complications is substantial.

The mechanisms involved in the evolution of HCV infection to LPDs are still not completely understood. The key role played by persistent HCV infection of the peripheral blood mononuclear cells (PBMCs) with a strong and sustained immune stimulation by HCV antigens, the cross-linking of CD81 by the HCV envelope protein E2 as well as the determination of t(14;18) translocation and/or other events inhibiting B-cell apoptosis, has been previously well documented [10], [13].

The development of MC and its evolution into a frank syndrome in only a portion of HCV-positive patients and the variable prevalence of this disease in different ethnic groups, strongly suggest that individual, genetic and epigenetic determinants are major contributions. This is corroborated by reports that have failed to identify specific viral variants associated with HCV-related LPDs [14], meaning that the host genetic background seems to play a pivotal role in determining susceptibility to MC in HCV subjects [15]. Specific HLA patterns closely associated with the development of MC have also been described [16].

In the last decade it has become evident that, together with genetic predisposition, epigenetic factors such as microRNA (miRNAs) expression can give an extremely important contribution to the pathogenesis of several diseases. The microRNAs are a class of endogenous 19-25-nucleotide long, non-coding RNA molecules regulating a wide variety of other genes by blocking the translation of complementary mRNAs. Therefore, miRNAs are important in a broad range of physiological and pathological processes and, as master modulators of gene expression, they are instrumental in regulating immune system development, normal immune functions and autoimmunity [17], [18], [19].

Interesting data demonstrated the essential role of liver-specific miRNA, miR-122, in HCV replication [20] and the miRNA pattern seems to be deregulated by HCV in liver cancer [21].

Modifications of miRNA expression pattern have been demonstrated for several types of human cancers [22], [23] as well as for a variety of autoimmune/lymphoproliferative disorders.

The number of miRNAs involved in the initiation and progression of human cancers is continuously increasing and such molecules have been designated as oncogenic miRNAs (onco-miRNAs) [24].

Since miRNAs control critical steps of the immune system and different stages of the hematopoietic maturation process, their misexpression is reputed to be the cause of several blood malignancies, correlating also with prognosis and phenotype. From the first evidence of the relevance of miRNAs in lymphoid tumors, several studies have confirmed the involvement of some cancer-related and newly identified miRNAs, namely miR-155, miR-146a, miR-21 and miR26b, in the pathogenesis of different subtypes of lymphomas including some HCV-related ones (marginal zone lymphoma –MZL and diffuse large B cell lymphoma -DLBCL) [25], [26], [27]. MiR-21 and miR-26b have been also associated with hepatocellular carcinoma development and worst outcome after liver cancer therapy, respectively [28], [29].

In the field of autoimmune diseases, the amplest data are available for rheumatoid arthritis (RA). Several reports have described altered miRNA expression in the synovium of patients with RA, indicating upregulation of miR-146a in human synovial tissue [30] and in the peripheral blood of RA patients, together with the upregulation of miR-155 and miR-16 [31]. The upregulation of miR-155, miR-146, miR-16 and miR-132 in synovial fibroblasts and PBMCs isolated from RA patients further confirms the involvement of miRNAs in the pathogenesis of such disorders [30], [31], [32]. The mechanisms through which these upregulated miRNAs contribute to RA pathogenesis are still not clear, but they are probably implicated in fuelling the chronic inflammatory processes.

Emerging evidence also exists for an increasingly interconnected network of miRNA dysregulation in systemic lupus erythematosus (SLE). Studies to date reveal a differential expression profile of several miRNAs in PBMCs, particularly in T cells isolated from SLE patients in comparison to healthy subjects (for a complete review [33]).

Although several unsolved issues about the specific mechanisms of action of miRNAs in the pathogenesis of such diseases need to be resolved, it is already clear that they have a huge potential as diagnostic and prognostic biomarkers of disease type and severity.

To date, very limited data exist about the miRNA expression pattern in HCV-related extrahepatic disorders and, in particular, no information is available about HCV-related MC. In this light, in the present study we evaluated the expression levels of a panel of selected miRNAs previously shown to be modified in autoimmune/lymphoproliferative disorders, in a large number of well characterized patients affected by HCV-related LPDs in order to identify miRNAs specifically modified in such disorders and possibly useful for developing diagnostic and/or therapeutic measures.

## Patients and Methods

### Patients

We studied 167 chronically HCV-infected patients, consecutively recruited from January 2009 to December 2010, with the following characteristics: 75 with MC [MC-HCV] (19 males, mean age 60.6±14.5 yr), 11 with HCV-associated NHL [NHL-HCV] (2 males, mean age 60.5±6.55 yr), 81 without MC or NHL or any signs or symptoms of autoimmune/lymphoproliferative disorder [HCV, pathological control] (60 males, mean age 55.1±12.1 yr), and 35 healthy blood donors (HS) as negative controls (Table 1). We also included 35 patients with HCV-unrelated liver disease (patients with chronic HBV hepatitis [34], consecutively recruited for anti-HBV treatment in the same period of time; 25 males, mean age 56.3±11.4 yr). The main clinical manifestations of MC-HCV patients are summarized in Table 2. The majority (8/11) of HCV patients with NHL also harbored MC and MC-related symptoms including purpura (75%), arthralgia (65%), peripheral neuropathy (53%), renal involvement (11%), sicca syndrome (20%), with frequency rates similar to MC patients without NHL.

**Table 1:** Main clinical and laboratory data of 35 healthy subjects (HS) and 167 HCV-chronically infected patients with mixed cryoglobulinemia (MC-HCV) or HCV-related non-Hodgkin’s lymphoma (NHL-HCV) or without MC or NHL (HCV).

	HS	HCV	MC-HCV	NHL-HCV	*p value*
	(n = 35)	(n = 81)	(n = 75)	(n = 11)	
**Mean Age** (years)	55±5.1	55.1±12.1	60.6±14.5	60.5±6.55	*p*<0.05*
**Sex** (male/female)	24/11	60/21	19/56	2/9	*p*<0.05**
**Histology**					*p*<0.05***
Chronic Hepatitis		66	47	9	
Cirrhosis		15	28	2	
Nodal marginal zone lymphoma				2	
Splenic marginal zone lymphoma				8	
Diffuse large B-cell lymphoma				1	
**ALT** ∧ (ULN)	0.69±1.3	1.68±1.19	1.07±0.6	1.54±0.5	ns
**Viral titer** (IU/mL x 10^6^)	–	3.0±3.9	3.5±7.4	3.1±2.2	ns
**HCV genotype**					ns
1		53	38	6	
2		14	33	3	
3		12	3	1	
4		2	1	1	
**Mean cryocrit** (%)	0	0	11.7±17.1	4.8±5.4	*p*<0.01°
**Mean C3** # (mg/dL)	132±31	108±23	91±24	89±17	ns
**Mean C4** ‡ (mg/dL)	126±16	105±27	8±7	6±6	*p*<0.01°
**Mean RF** † (IU/mL)	15±8	16±8	273±126	322±159	*p*<0.01°

Results are presented as mean± standard deviation;

ns, not significant;

∧ ALT, alanine aminotransferase;

ULN, upper limit of normal.

# Complement C3, normal values: 83 to 177 mg/dL;

‡ Complement C4, normal values: 20 to 150 mg/dL;

† Rheumatoid Factor, normal values: <25 IU/mL.

* HS vs MC-HCV; HS vs NHL-HCV; HCV vs MC-HCV.

** HS vs MC-HCV; HS vs NHL-HCV; HCV vs MC-HCV; HCV vs NHL-HCV.

*** HCV vs MC-HCV.

°HS or HCV vs MC-HCV or NHL-HCV.

**Table 2:** Principal mixed cryoglobulinemia (MC) manifestations present in the 75 HCV patients with MC (MC-HCV).

MC manifestations	Patients	(%)
	(n = 75)	
**Purpura**	58	77.3
**Arthralgias**	62	82.7
**Weakness**	69	92
**Neuropathic symptoms**	52	69.3
**Renal involvement**	11	14.7
**Skin ulcers**	8	10.7
**Sicca syndrome**	25	33.3

The MC-HCV group included a subgroup of 35 patients (5 males, mean age 52.9±5.2 yr) also belonging to another study (Giannini *et al*, manuscript in preparation) analyzing the long-term behavior of MC-HCV patients undergoing sustained virological response to antiviral therapy with pegylated interferon and ribavirin according to standard protocols [35], [36]. This subgroup of patients experienced both complete viral eradication and MC clinical response. Biological samples from these patients were analyzed both before treatment and 6 months after the end of therapy. HCV infection was proven by detecting circulating anti-HCV antibodies (EIA-2 and RIBA-2, Ortho Diagnostic Systems, Raritan, NJ) and HCVRNA (AMPLICOR® HCV Test, v2.0. Roche Diagnostics, Alameda, CA). HCV genotype was determined by a commercial, certified, diagnostic test (VERSANT HCV Genotype 2.0, Siemens Healthcare Diagnostics, Deerfield, IL). Liver disease was diagnosed according to standard, previously described criteria and was based on liver biopsy [37]. Histopathology of liver samples was assessed by two independent expert pathologists external to the study, using the METAVIR algorithm [37].

MC was diagnosed according to previously described criteria [38]. Serum cryoglobulin levels and characterization, complement fraction levels, rheumatoid factor (RF) and autoantibodies were evaluated as described [38], [39], [40]. In particular, MC patients had detectable serum cryoglobulins for more than 6 months and at least 2 of the following criteria: palpable purpura, positive IgM-RF latex test and low C4 levels.

According to treatment inclusion criteria [41] patients had mild to moderate MC syndrome; patients with severe/life threatening MC syndrome were excluded.

A diagnosis of splenic marginal zone B-cell lymphoma (SMZL), nodal marginal zone lymphoma (NMZL), and diffuse large B-cell lymphoma (DLBCL) was done in 8, 2, and the remaining NHL patient, respectively, according to the Revised European American Lymphoma Classification (REAL) [42]. The blood sampling was performed at the diagnosis of lymphoma, and before treatment.

All the patients included in this study provided written informed consent in accordance with the principles of the Declaration of Helsinki and the study was approved by the University of Florence Ethics Committee.

### Cell Isolation

Peripheral blood mononuclear cells (PBMCs) were isolated from fresh anticoagulated blood by gradient precipitation on Lymphoprep (Axis-Shield PoC AS, Oslo, Norway) according to the manufacturer’s instructions. After the second wash, the cells were counted and stored at −80°C. The mean storage time was 27.35±7.12 months.

### RNA Extraction

Total RNA was extracted using Trizol (Invitrogen, USA) from 5×10^6^ purified PBMCs according to the manufacturer’s instructions. *C. elegans* miR-39 synthetic RNA oligonucleotide (1.1×10^8^ copies/10^6^ cells) was added to PBMC samples and used as external control to monitor extraction efficiency.

### Reverse Transcription and Quantitative Real-time PCR for miRNA Expression

Reverse transcription (RT) was performed using TaqMan MicroRNA RT kit (Applied Biosystems, CA, USA) and 160 nanograms of total RNA.

Expression levels of human miR-Let-7d, miR-16, miR-21, miR-26b, miR-146a, miR-155 and *C. elegans* miR-39 were evaluated by Real Time PCR using specific TaqMan MicroRNA Assays (Applied Biosystems, CA, USA) according to the manufacturer’s instructions.

Relative expression levels of the different miRNAs were evaluated by using the 2^−ΔΔct^ method [43], with miR-Let-7d as internal control to normalize miRNA expression levels.

### Statistical Analysis

Data are expressed as the mean ± SD. Quantitative variables were analyzed using one-way analysis of variance (non-parametric ANOVA). Categorical variables were analyzed with the χ^2^ test and Fisher’s exact test when necessary. All tests were two-sided at a 0.05 significance level. Analyses were performed by the Stata v.9.0 (StataCorpLP, College Station, TX, USA).

## Results

The main clinical and serological data of these patients are reported in Table 1. The studied groups did not significantly differ in terms of viremia titers or viral genotype distribution, whereas, as expected, the female sex was significantly more represented in the MC-HCV group (*p*<0.05), as well as older age (*p*<0.01) and liver cirrhosis (*p*<0.05) (Table 1).

**Figure 1:**
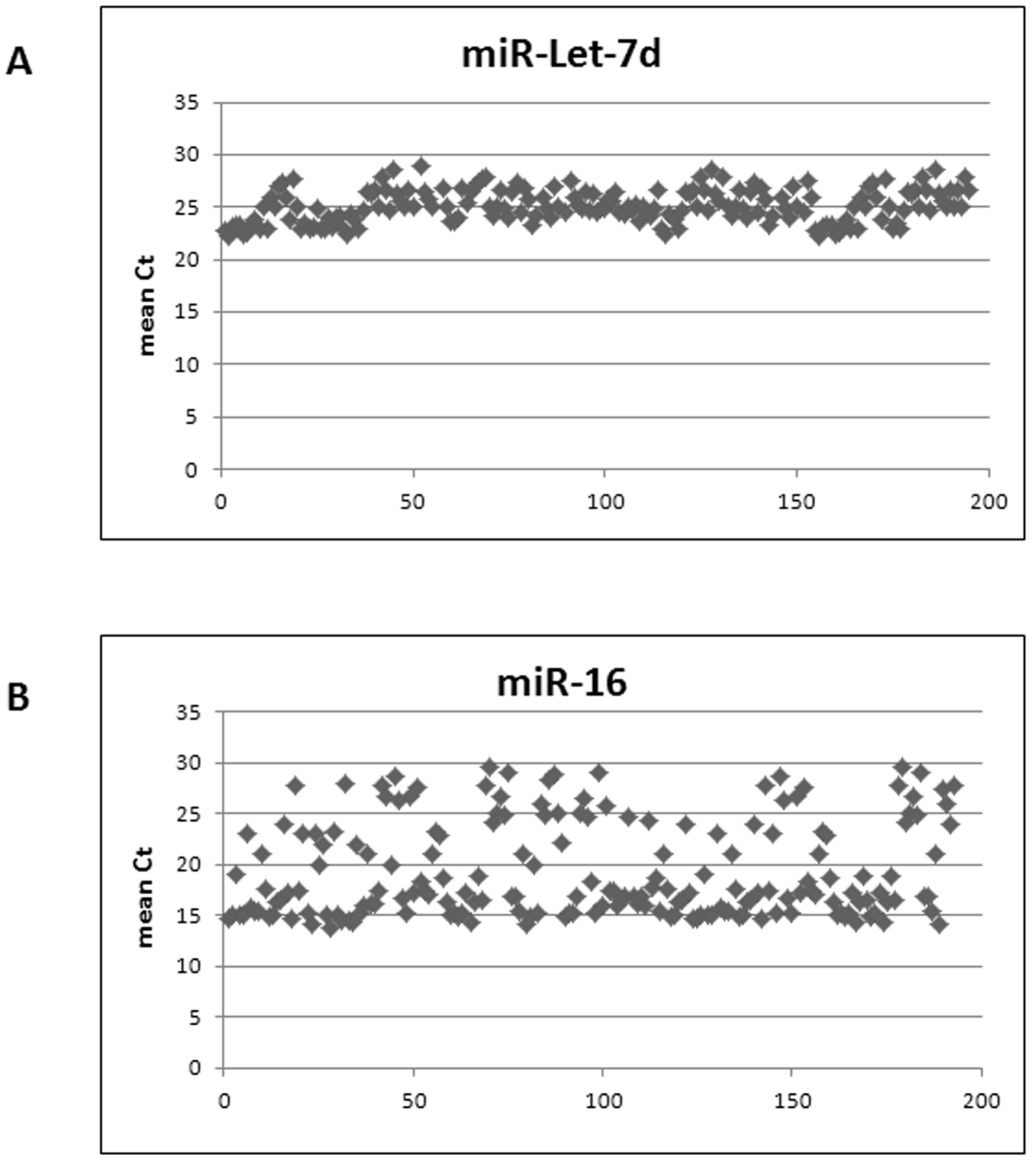
Identification of a reference miRNA (internal control) for relative quantification of miRNA of interest: expression levels of Let-7d (panel A) and miR-16 (panel B) in HCV patients and controls.

The expression levels of Let-7d resulted stable in all subjects analyzed in the study (mean Ct: 25.15±0.85, Figure 1, panel A). Conversely, miR-16 levels showed a higher level of fluctuation (mean Ct: 19.33±4.61, Figure 1, panel B).

**Figure 2:**
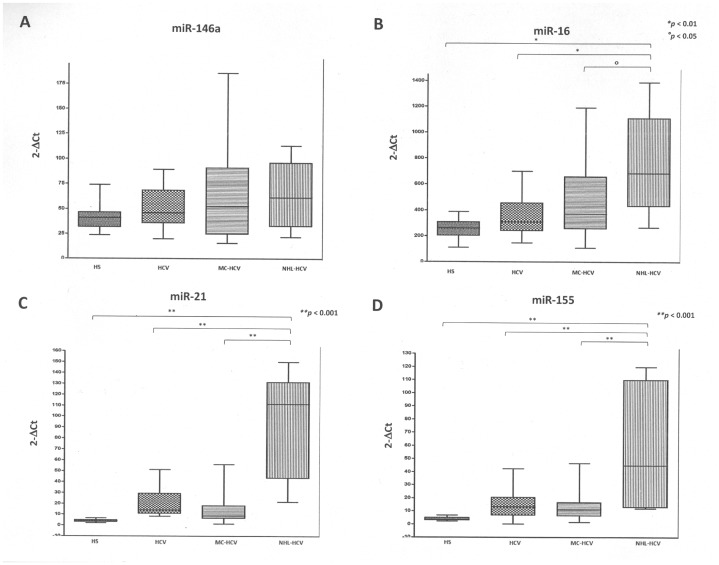
Expression levels of analyzed miRNA in healthy subjects (HS), HCV, MC-HCV and NHL-HCV patients: miR-146a (panel A), miR-16 (panel B), miR-21 (panel C) and miR-155 (panel D).

The expression levels of miR-146a were similar in all tested groups although a higher range of distribution of the samples appeared for MC-HCV patients (Figure 2, panel A).

MiR-16, miR-21 and miR-155 had similar expression profiles among the studied groups. In fact, a significantly higher level of these miRNAs was present in the PBMCs isolated from NHL-HCV patients when compared to all other groups. In more detail, miR-16 levels were 3.16 and 2.15 times more expressed in NHL than in HS and HCV individuals, respectively (*p*<0.01), and 1.54 times vs MC-HCV patients (*p*<0.05). MiR-21 showed a 24.51-, 4.34-, 6.98-fold induction in NHL vs HS, HCV and MC-HCV individuals, respectively (*p*<0.001). Analogously, miR-155 was up-regulated 13.12-, 3.68- and 3.79-times in NHL vs HS, HCV and MC-HCV patients, respectively (*p*<0.001).

No significant differences in miR-16, miR-21 and miR-155 levels of expression were evident in HS, HCV and MC-HCV groups.

**Figure 3:**
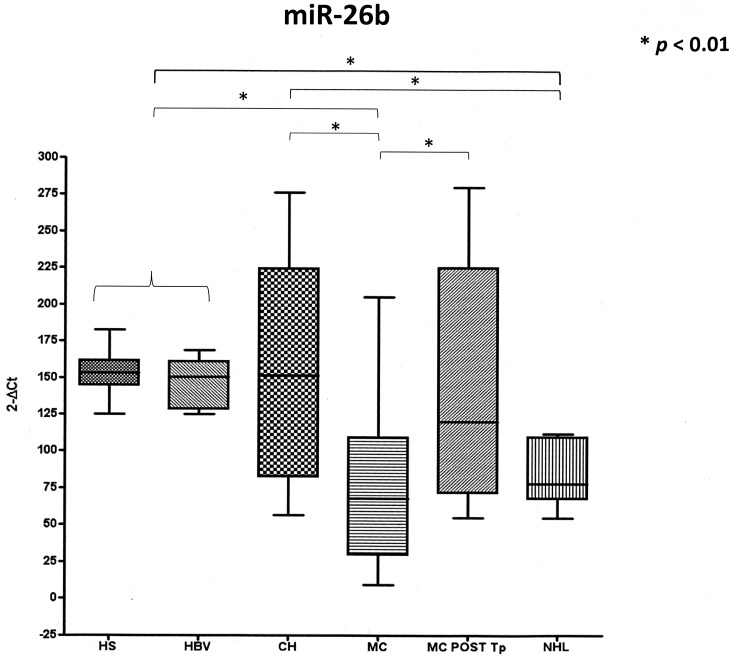
Expression levels of miR-26b in HS, HBV, HCV, MC-HCV and NHL-HCV groups and in a subgroup of MC-HCV patients after therapy-induced clearance of the viral infection and complete clinical response (MC post Tp).

Interestingly, when we considered the miR-26b, a strong down-regulation was detected in MC and NHL groups when compared to HS, HBV and HCV patients, who presented similar levels (74±51.14 and 83.61±23.36 vs 162.48±11.28, 160.13±18.56 and 151.98+75.44, respectively; *p*<0.01). In the MC-HCV group, samples obtained from 35 patients before and >6 months after therapy-induced complete clearance of the viral infection and complete clinical response were available. The concentration of miR-26b in these latter samples was totally restored (144.05±80.21) to levels comparable to HS, HBV patients or patients without LPD (HCV) (Figure 3).

## Discussion

In the present study, for the first time, we analyzed modifications in the expression pattern of some miRNAs in PBMCs from patients with HCV-positive LPDs (MC and NHL). A panel of miRNAs previously shown to be involved in autoimmune and/or lymphoproliferative disorders sharing analogies with HCV-related diseases (i.e, rheumatoid arthritis, Sjogren syndrome, MZL, DLBCL) was selected in order to identify miRNAs with a potential value as diagnostic/prognostic markers.

A critical step in the relative quantification of miRNA expression levels is represented by the selection of a miRNA that is constantly expressed in patients and controls. This issue is currently disputed and housekeeping candidates vary, depending on the tissue/cell type analyzed. Several studies have been conducted using the miR-16 as internal reference [44]. However, in the present study, miR-16 expression was significantly different in the NHL group compared to the others (Figure 2, panel B). Conversely, miR-Let-7d showed constant and homogeneous expression levels in PBMCs from all subjects analyzed and assured a good estimation of the levels of the miRNA of interest. Consistently, Qi *et al.* also observed a higher reliability of miR-Let-7d as internal control [45].

Concerning the analysis of miRNA expression, miR-146a levels did not significantly differ in MC or NHL patients compared to controls, in spite of previous reports describing a misregulation of miR-146a in several autoimmune disorders, namely AR [31] and LES [46].

A common expression profile was found for three miRNAs frequently associated with neoplastic disorders (onco-miRNAs): miR-16, miR-21, and miR-155. In fact, these miRNAs were up-regulated in PBMCs from NHL patients, whereas comparable expression levels were recorded in HCV, MC and healthy controls. This observation suggests the potential utility of the analysis of the onco-miRNA expression profile in HCV patients with suspected lymphomatous evolution.

In more detail, miR-21 has been previously described as dramatically up-regulated in cancers, specially solid, of various origin [47]. The identified target genes of miR-21 are numerous and mostly involved in cell cycle regulation and suppression of growth and proliferation and, according to computational analysis, several other targets remain to be identified [47]. The upregulation of miR-21 in HCV-associated lymphoma patients we describe in the present study is consistent with recent data concerning splenic SMZL [26].

MiR-155 is another very interesting miRNA involved in the pathogenesis of several autoimmune disorders [48] as well as different types of lymphoma [49], [50]. Previous reports have indicated miR-155 as a regulator of activation-induced cytidine deaminase (AID), a potent DNA mutator that has been involved in HCV-mediated lymphomagenesis [51], [52]. These data justify the analysis of miR-155 expression also in HCV-related LPDs. Higher levels of miR-155 were evident only in NHL patients, a result consistent with previously published data obtained in lymphoma tissue. However, here we show that it is possible to reproduce similar results also in PBMC samples with an evident translational potential.

Finally, of special interest was the result of miR-26b analysis, showing a statistically significant downregulation in both MC and NHL patients compared to controls. A recent study, focused on HCV-positive SMZL, reported an association between this specific LPD and miR-26b downregulation in lymphoma tissue samples, suggesting the involvement of miR-26b downregulation in HCV-associated lymphomagenesis [26]. Our present results confirm and consistently expand these previous data showing the involvement of such a miRNA, not only in the specific field of HCV-associated SMZL, but also in MC, the most frequent HCV-related extrahepatic disorder, as well as in different HCV-associated NHLs including first MZL and also DLBCL. In addition, the present study shows the interest of the determination of miRNA expression pattern in PBMCs, obtainable through simple, non-invasive blood sampling, thus consistently amplifying the translational potential of such a correlation. A deeper analysis of the mononuclear cell sub-populations is ongoing and will clarify the actual contribution to miRNA deregulation of the different cell types.

Due to the complex and variable pattern of miRNA expression, we introduced an additional control represented by PBMC samples taken from the same MC patients before and after complete HCV clearance and complete clinical response according to previously shown criteria [53], [54]. In fact, after the complete virological and clinical response to the anti-HCV therapy, in MC patients the downregulation of miR-26b determined before treatment was no longer observed. Conversely, MC patients who did not respond to the therapy and maintained HCV viremia and MC symptoms after a 6-months washout period, showed miR-26b expression levels comparable with pre-treatment values and with the levels detected in MC untreated population (data not shown). The dowregulation of miR-26b in HCV patients with LPDs, and not in HCV patients without LPDs, before and after therapy, suggests that the evolution of the infection to LPDs -instead of HCV infection *per se* is critical. Consequently, it is conceivable that modifications of miR-26b expression could be useful to monitor the development of MC or NHL during the course of HCV infection. However, further analysis of larger populations of selected patients are needed to ascertain such hypothesis.

Actually, an interesting, still unresolved, issue concerns the mechanisms triggered by miR-26b deregulation. A possible role can be played by the miR-26b target, Nek6, coding for a kinase involved in the initiation of mitosis; the overexpression of Nek6, already observed in different tumors [55], [56], could contribute to the avoidance of cellular senescence and promote cancer progression [26]. In addition, other miR-26b targets may help promote and/or sustain lymphomagenesis, i.e. lymphoid enhancer factor 1 (LEF-1). LEF-1 is a nuclear transcription factor which forms a complex with β-catenin and T-cell factor and induces transcription of cyclin D1 and c-myc; it is transiently expressed in the pro-B cells while it is not detectable in mature normal B lymphocytes. LEF-1 has been recently indicated as a specific target of miR-26b and its overexpression is associated with miR-26b downregulation in different cell line models [57]. Several studies have shown elevated levels of LEF-1 in human cancers, in particular in chronic lymphatic leukemia (CLL) [58] and a very recent study describes an alteration in LEF-1 mRNA and protein expression in a wide cohort of DLBCLs [59]. The possible involvement of LEF-1 in the pathogenesis of HCV-related LPDs is currently under evaluation.

In conclusion, this study strongly suggests the relevance of modifications of miRNA expression pattern in the pathogenesis of HCV-related LPDs and the utility of miRNAs as potential markers of disease evolution/progression from pre-neoplastic conditions to the frank malignancy. Further prospective studies will be useful to ascertain these hypotheses.
